# A causal relationship between cigarette smoking and type 2 diabetes mellitus: A Mendelian randomization study

**DOI:** 10.1038/s41598-019-56014-9

**Published:** 2019-12-18

**Authors:** Shuai Yuan, Susanna C. Larsson

**Affiliations:** 1 Unit of Cardiovascular and Nutritional Epidemiology, Institute of Environmental Medicine, Karolinska Institutet, Stockholm, Sweden; 2 Department of Surgical Sciences, Uppsala University, Uppsala, Sweden

**Keywords:** Type 2 diabetes, Risk factors

## Abstract

The causality between smoking and type 2 diabetes is unclear. We conducted a two-sample Mendelian randomization study to explore the causal relationship between smoking initiation and type 2 diabetes. Summary-level data for type 2 diabetes were obtained from a meta-analysis of 32 genome-wide association studies (DIAbetes Genetics Replication And Meta-analysis consortium), which included 898 130 individuals of European ancestry. Totally, 377 single-nucleotide polymorphisms associated with smoking initiation at genome wide significance threshold (*p* < 5 × 10^−8^) were identified from the hitherto largest genome-wide association study on smoking. The inverse-variance weighted, weighted median, MR-Egger regression, and MR-PRESSO approaches were used to analyze the data. Genetically predicted smoking initiation was associated with type 2 diabetes with an odds ratio of 1.28 (95% confidence interval, 1.20, 1.37; *p* = 2.35 × 10^−12^). Results were consistent across sensitivity analyses and there was no evidence of horizontal pleiotropy. This study provides genetic evidence supporting a causal association between the smoking initiation and type 2 diabetes. Reducing cigarette smoking initiation can now be even more strongly recommended for type 2 diabetes prevention.

## Introduction

Globally, type 2 diabetes mellitus is a public health issue, affecting around 1 in 11 adults in 2015^1^. Cigarette smoking has been proposed as an independent and modifiable risk factor for type 2 diabetes in observational studies among both men and women^2–4^. Results of a recent meta-analysis of 88 observational prospective studies, including 5 898 795 participants and 295 446 incident cases of type 2 diabetes, showed that current smoking was associated with a 37% increased risk of developing type 2 diabetes compared with non-smoking^3^. A dose-response relationship was identified across light, moderate and heavy smokers^3^. Nevertheless, whether the association between smoking and type 2 diabetes is causal remains unclear due to potential residual confounding and reverse causality that could bias the results from observational studies. In addition, the causality is not feasible to be tested in the experimental settings considering the ethical issues.

Mendelian randomization (MR) is a technique that minimizes unobserved confounding and reverse causality by proposing genetic variants as instrumental variables of an exposure^5^. Genetic variants have no relevance with self-adapted lifestyle factors and behaviors as they are randomly assorted at meiosis. Reverse causality can be eliminated as allelic randomization precedes disease onset. Therefore, we conducted a MR study to assess the associations between smoking initiation and risk of type 2 diabetes.

## Methods

### Study design overview

We employed an MR design to investigate the association between smoking initiation and type 2 diabetes using summary-level data from two large genome-wide association studies for tobacco use and type 2 diabetes. Details of used datasets are shown in Supplementary Table 1. There are three key assumptions for an MR study^6^. These include: (1) the genetic variants selected as instrumental variables should be strongly associated with the exposure; (2) the instrumental variables should not be associated with confounders of the exposure-outcome association; (3) the instrumental variables should affect the risk of the outcome merely through the risk factor, not via alternative pathways. Ethical approval for this study was obtained from the Swedish Ethical Review Authority. This MR study only uses published or publicly available summary-level data (i.e., beta coefficients and standard errors). Ethical approval (and informed consent from each participant) for each study included in the genome-wide association studies of tobacco use and type 2 diabetes can be found in the original articles^7,8^. All procedures performed in studies involving human participants were in accordance with the ethical standards of the institutional or national research committee and with the 1964 Helsinki declaration.

### Outcome sources and SNP selection

Summary-level data for type 2 diabetes were obtained from a publicly available genome-wide association study (GWAS) of 32 studies (DIAbetes Genetics Replication And Meta-analysis consortium), which included 898 130 individuals (74 124 cases and 824 006 controls) of European ancestry^7^. A harmonized protocol was developed to improve the quality of the genotype scaffold in each study. The HRC reference panel was used in the imputation stage and adjustments were made for population structure (e.g., through principal components), relatedness and study-specific covariates. Data without body-mass index adjustment were used in the main analysis, and data adjusted for body-mass index were used in the sensitivity analysis in the present study.

Selection of instrumental variables was based on a recent published meta-analysis of GWASs for smoking initiation, which included data from up to 1 232 091 individuals of European ancestry^8^. In total, 378 conditionally independent single-nucleotide polymorphisms (SNPs) associated with smoking initiation at the genome wide significance threshold (*p* < 5 × 10^−8^) were identified^8^ of which all but one SNP was available in the type 2 diabetes dataset. Adjustments had been made for genetic principal components^8^. Detailed information for each SNP is shown in Supplementary Table 2. Smoking initiation was defined as having smoked >100 cigarettes over the course of your life, smoked every day for at least a month or ever smoked regularly. The GWAS for smoking initiation reported the effect sizes in the unit of standard deviation, which was calculated from the weighted average prevalence across all included studies^8^.

### Statistical analyses

The association between genetically predicted smoking initiation and type 2 diabetes attributable to each SNP was estimated with the Wald method, which computes the ratio between the SNP-diabetes and SNP-smoking estimates. In the main analysis, the ratio estimates for individual SNPs were combined by using the multiplicative random-effects inverse-variance weighted meta-analysis method^9^. We also performed supplementary analysis based on the weighted median, MR-Egger (with or without adjustment via Simulation Extrapolation [SIMEX] method), MR-PRESSO approaches, to examine the robustness of the association and assess whether the MR assumption of no pleiotropy is met. The inverse-variance weighted method provides the most precise estimates but could be influenced by invalid instrumental variables and pleiotropic effects^9^. The weighted median approach provides a consistent estimate under the requirement that more than half of the weight in the analysis comes from valid instrumental variables^10^. The MR-Egger method identifies and corrects for directional pleiotropy, albeit with low power^11^. MR-Egger regression with SIMEX adjustment was used as a sensitivity analysis as regression dilution bias in the SNP-smoking estimates was observed in the standard MR-Egger analysis (I^2^_GX_ < 90%)^12^. The MR pleiotropy residual sum and outlier (MR-PRESSO) test aims at detecting possible outliers and results obtained from the MR-PRESSO analysis are corrected for horizontal pleiotropy via outlier removal^13^. Rucker’s Q’ value was additionally estimated to measure the heterogeneity in the MR-Egger analysis and as a comparison with Cochran’s Q value. A Rucker’s Q’ value that is lower than Cochran’s Q value indicates that the MR-Egger method provides a model with better fit for examining the particular association.

We harmonized the summary statistics data across datasets so that the effect allele reflected the allele associated with an increased probability of lifetime smoking initiation. When SNPs were palindromic (i.e., A/T or G/C), we used information on allele frequency to resolve strand ambiguity. Ten palindromic SNPs with minor allele frequency above 0.45 were retained because the SNPs were read from the same strand, and exclusion of those ten palindromic SNPs did not change the results. We estimated mean F-statistics to assess the strength of the instrumental variables^14^ and got an F-statistic of 76.7. The reported odds ratios (ORs) and confidence intervals (CIs) of type 2 diabetes correspond to the increase of one standard deviation in prevalence of smoking initiation. All estimates are reported with two-tailed P values. The statistical analyses were performed in Stata/SE 15.0 using the mrrobust package^15^, except the MR-PRESSO analysis which was conducted in R software 3.6.0.

## Results

**Figure 1:**
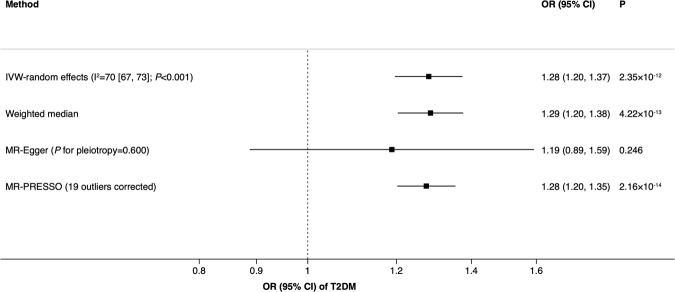
Association between smoking initiation and type 2 diabetes from Mendelian randomization. CI indicates confidence interval; OR, odds ratio; T2DM, type 2 diabetes mellitus.

Genetically predicted smoking initiation was positively associated with type 2 diabetes. The ORs of type 2 diabetes were 1.28 (95% CI, 1.20, 1.37; *p* = 2.35 × 10^−12^) and 1.29 (95% CI, 1.20, 1.38; *p* = 4.22 × 10^−13^) in the inverse-variance weighted and weighted median models, respectively (Fig. 1). There was substantial heterogeneity across estimates of included SNPs with an I^2^ value of 70% (95% CI 67%, 73%) and a Cochran’s Q value of 1255 (*p* < 0.001). The MR-Egger estimate without SIMEX adjustment was directionally consistent with the other estimates, albeit non-significant with a wide CI. We observed an I^2^_GX_ of 61%, indicating dilution of the MR-Egger estimate due to violation of the No Measurement Error assumption. In a sensitivity analysis to correct for dilution bias using SIMEX adjustment, the OR was 1.22 (95% 0.72, 2.09; *p* = 0.457). There was no indication of horizontal pleiotropy in the MR-Egger analysis (intercept 0.001; 95% CI −0.004, 0.007; *p* = 0.600). We observed a Rucker’s Q’ value of 1254 (*p* < 0.001), indicating that the MR-Egger approach did not provide a model with better fit compared to the inverse-variance weighted method. In the MR-PRESSO analysis, we detected 19 possible outliers. After outlier correction, the OR of type 2 diabetes was 1.28 (1.20, 1.35; *p* = 2.16 × 10^−14^) (Fig. 1). A scatter plot for the associations of the smoking-related SNPs with smoking initiation and type 2 diabetes is shown in Supplementary Fig. 1. Results remained in the same pattern in the sensitivity analysis based on data with body mass index adjustment (Supplementary Table 3).

## Discussion

Findings of the present study support a causal association between smoking initiation and increased risk of type 2 diabetes. Most available studies found a consistent detrimental effect of current and past smoking on type 2 diabetes^2–4^, which is in line with the present study. A systematic review including 1.2 million participants revealed a dose-response relationship between active smoking and risk of type 2 diabetes among the pooled whole population and all subgroups^2^. Although several large-scale studies indicate that smoking cessation increases the short-term risk of type 2 diabetes, which is mediated by body mass index, the risk decreases substantially with time since quitting smoking^3,16^.

The mechanisms behind the causality between smoking and type 2 diabetes are not fully understood. There are several plausible explanations, such as negative effects of cigarette smoking on the function^17^ and mass of islet β-cells^18^, gastrointestinal tract^19^, nervous system^20,21^, obesity^22^ and inflammation^23^. Nicotine, a major bioactive element of cigarette, has been proved to impair the function and mass of the islet β-cells^17,18^, thereby disturbing its feedback regulation and interrupting glucose homoeostasis, which plays an important role of type 2 diabetes onset^24^. Smoking also negatively influences the function of gastrointestinal tract^19^, such as suppressing bile acids, which is of great importance in the regulation of glucose metabolism^24^. Recently, smoking has been found to be associated with change of composition of intestinal microbiome^25^ that potentially acts as a vital part in the pathophysiology of type 2 diabetes^24^. In addition, cigarette smoking also influences the function of the nervous system, such as vagus^20^, hypothalamus^21^, and circadian rhythmicity^26^, which are important regulators of glucose metabolic processes^24^. Inflammation induced by smoking also partly explains the causality^23^. Proinflammatory factors and C-reactive protein overproduction is associated with insulin resistence, β-cells function impairment and metabolism-related neuronal injury^24^. Even though these pathological pathways have been established to explain the causality between smoking and type 2 diabetes, more investigations on its etiology are needed, especially from genetics, epigenetics and omics^24^, for type 2 diabetes prevention and treatment.

A major limitation is that the dose-response relation of smoking heaviness (e.g., the number of cigarettes smoked per day) with type 2 diabetes could not be assessed in the present study because we could not exclude never smokers based on summary-level data. Another limitation is that there was a large overlap of participants included in the datasets for smoking initiation and type 2 diabetes, potentially leading to bias in the causal estimate in the direction of the observational association between smoking initiation and risk of type 2 diabetes^27^.

The validity of the results of an MR study depends on whether the MR assumptions are met. In this study, we only used SNPs that are strongly associated with smoking initiation at the genome-wide significance level, thereby reducing possible violation of the first assumption. A strong risk factor and thus a potential confounder in analyses of type 2 diabetes is body mass index. A positive association between smoking and type 2 diabetes remained when using data with body mass index adjustment. In addition, the consistency across sensitivity analyses indicated a negligible distortion by potential pleiotropy.

## Conclusion

This study provides genetic evidence supporting a positive causal association between the smoking initiation and type 2 diabetes. Thus, by more firm scientific support, reducing cigarette smoking initiation for type 2 diabetes prevention can now be even more strongly recommended. The mechanisms behind the relationship warrant more investigations.

## Supplementary information

Supplementary material

## Data Availability

All summary-level data necessary to conduct this MR analysis are included in Supplementary Table 2.
