# Supplementary material for: A causal relationship between cigarette smoking and type 2 diabetes mellitus: A Mendelian randomization study
Source: Sci Rep. 2019 Dec 18;9:19342. doi: 10.1038/s41598-019-56014-9 (PMC6920406; doi:10.1038/s41598-019-56014-9)
Supplement: Supplementary file 1 — Supplementary material [file 41598_2019_56014_MOESM1_ESM.pdf]

SUPPLEMENTARY MATERIAL

A causal relationship between cigarette smoking and type 2 diabetes mellitus: A Mendelian randomization study

Shuai Yuan[1,2] & Susanna C. Larsson[1,2]

[1]Unit of Cardiovascular and Nutritional Epidemiology, Institute of Environmental Medicine, Karolinska Institutet, Stockholm, Sweden

[2]Department of Surgical Sciences, Uppsala University, Uppsala, Sweden

Supplementary Table 1. Details of studies and datasets used for analyses

Exposure/ Outcome	Consortium or cohort study	Participants	Web source if publicly available
Smoking	Add Health, ALSPAC, ARIC, BBJ, BEAGESS, BLTS, CADD, CPGEND, COPDGene, deCODE, EGCUT, MERGE, Finntwin & NAG-FIN, FHS, GERA, GfG, HUNT, HRS, MCTFR, MESA, METSIM, NESCOG, NHS, NHS2, HPFS, NINDS SiGN, NTR, OZALC, SardiNIA, UK Biobank, WHI	1 232 091 million individuals of European ancestry	https://conservancy.umn.edu/handle/11299/201564
Type 2 diabetes	BioMe, deCODE, DGDG, DGI, GCUT_ExomeCore, EGCUT_Human370CNV, EGCUT_OmniExpress, FHS, FUSION, GCKD, GENOA, GERA, G0DARTS, GOMAP-TEENAGE, HPFS, INTERACT_GWAS, KORA, MESA, METSIM, MGI, NHS, NUGENE, PIVUS, PROSPER, RS1, RS2, RS3, UK Biobank, ULSAM, UPCH, WTCCC	898 130 individuals (74 124 type 2 diabetes cases and 824 006 controls) of European ancestry	http://diagram-consortium.org/downloads.html

Add Health indicates National Longitudinal Study of Adolescent to Adult Health; ALSPAC, Avon Longitudinal Study of Parents and Children; ARIC, Atherosclerosis Risk in Communities; BBJ, BioBank Japan Project; BEAGESS, The Barrett's and Esophageal Adenocarcinoma. Genetic Susceptibility Study; BioME, BioMe™ BioBank Program; BLTS, Brisbane Longitudinal Twin Study; CADD, Center on Antisocial Drug Dependence; COGEND, Collaborative Genetic Study of Nicotine Dependence; COPDGene, Genetics of Chronic Obstructive Pulmonary Disease; deCODE, deCODE Genetics/AMGEN, Inc.; DGDG, Diabetes Gene Discovery Group; DGI, Diabetes Genetics Initiative; EGCUT, Estonian Genome Center; eMERGE, Electronic Medical Records and Genomics; Finntwin & NAG-FIN, Finnish Twin Cohort; FHS, Framingham Heart Study; FUSION, Finland-United States Investigation of NIDDM Genetics; GCKD, German Chronic Kidney Disease Study; GENOA, Genetic Epidemiology Network of Arteriosclerosis; GERA, Genetic Epidemiology Research in Adult Health and Aging; GfG, Genes for Good; GoDARTS, Genetics of Diabetes and Audit Research in Tayside Scotland; GOMAP-TEENAGE, GoMAP (Genetic Overlap between Metabolic and Psychiatric traits) & TEENAGE (TEENs of Attica: Genes and Environment); HUNT, The Nord-Trøndelag Health Study; HPFS, Health Proffesional Follow-Up Study; HRS, Health and Retirement Study; INTERACT_coreexome, InterAct Consortium; INTERACT_GWAS, InterAct Consortium; KORA, KORAgen Study Helmholtz zentrum München; MCTFR, Minnesota Center for Twin and Family Research; MESA, Multi-Ethnic Study of Atherosclerosis; METSIM, Metabolic Syndrome in Men; MGI, Michigan Genomics Initiative; NESCOG, Netherlands Study on Cognition, Environment and Genes; NHS, NHS2, and HPFS, Nurses' Health Study, Nurses' Health Study II, and Health Professionals' Follow-up Study; NINDS SiGN, The National Institute of Neurological Disorders and Stroke Genetics Network; NTR, Netherlands Twin Register; NUGENE, NUgene Project; OZALC, Australian Twin-Family Studies on Nicotine and Alcohol Genetics; PIVUS, Prospective Investigation of the Vasculature in Uppsala Seniors; PROSPER, PROspective Study of Pravastatin in the Elderly at Risk; RS1, RS2, RS3, Rotterdam Study; SardiNIA, SardiNIA project; ULSAM, Uppsala Longitudinal Study of Adult Men; UPCH, Danish T2D case-control study; WHI, Women's Health Initiative; WTCCC, Wellcome Trust Case-Control Consortium.

Supplementary Table 2. Characteristics of the single-nucleotide polymorphisms associated with smoking initiation and its associations with type 2 diabetes

				Smoking initiation						Type 2 diabetes			
**SNP**	**Chr**	**Closest Gene**	**EA**	**OA**	**EAF**	**Beta**	**SE**	**P**		**EAF**	**Beta**	**SE**	**P**
rs1008078	1	Intergenic	T	C	0.40	0.023	0.003	1.63E-18		0.40	0.009	0.007	0.150
rs1022528	1	Intron:PTGER3	A	G	0.34	0.017	0.003	8.48E-11		0.35	0.009	0.007	0.180
rs10789369	1	Intergenic	A	G	0.39	0.023	0.003	3.39E-19		0.38	-0.010	0.007	0.120
rs10873871	1	Intron:ST6GALNAC3	G	A	0.21	0.018	0.003	2.82E-08		0.21	0.014	0.008	0.077
rs10914684	1	Intron:PHC2	G	A	0.68	0.016	0.003	6.32E-09		0.67	0.002	0.007	0.760
rs11162019	1	Intergenic	C	T	0.64	0.016	0.003	5.06E-09		0.64	0.001	0.007	0.880
rs11587399	1	Intergenic	A	T	0.78	0.018	0.003	7.25E-09		0.78	0.029	0.008	0.000
rs12022778	1	Intron:ELAVL4	C	A	0.20	0.027	0.003	3.18E-17		0.20	-0.004	0.008	0.600
rs12027999	1	Intron:UBAP2L	T	C	0.88	0.024	0.004	5.33E-10		0.87	-0.023	0.010	0.016
rs12130857	1	Intron:CAMTA1	G	A	0.68	0.018	0.003	3.65E-11		0.68	0.007	0.007	0.310
rs12563365	1	Intron:ACTN2	A	G	0.56	0.017	0.003	1.05E-10		0.55	0.010	0.006	0.110
rs12739243	1	Intron:SYT14	T	C	0.78	0.021	0.003	4.45E-12		0.77	0.021	0.008	0.005
rs12740789	1	Intergenic	G	A	0.82	0.029	0.003	1.18E-17		0.81	0.040	0.008	0.000
rs12755632	1	Intergenic	A	G	0.68	0.015	0.003	1.93E-08		0.68	0.014	0.007	0.047
rs147052174	1	Nonsynonymous:FAM163A	T	G	0.02	0.062	0.010	2.30E-10		0.02	0.083	0.026	0.001
rs1514176	1	Intron:FPGT-TNNI3K|TNNI3K	G	A	0.42	0.019	0.003	7.67E-14		0.43	-0.003	0.006	0.640
rs1889571	1	Intron:ADGRB2	G	T	0.13	0.022	0.004	4.19E-09		0.13	0.019	0.010	0.049
rs1935571	1	Intergenic	T	G	0.52	0.016	0.003	6.99E-10		0.52	0.015	0.006	0.021
rs1937443	1	Intron:PDE4B	G	C	0.56	0.020	0.003	1.79E-15		0.57	0.007	0.007	0.330
rs2637869	1	Intergenic	A	G	0.30	0.018	0.003	6.54E-11		0.30	-0.013	0.007	0.070
rs2901785	1	Intron:LOC102724601	G	A	0.55	0.017	0.003	1.47E-11		0.57	-0.001	0.006	0.940
rs301807	1	Intron:LOC102724552|RERE	G	A	0.57	0.018	0.003	2.50E-12		0.58	-0.002	0.006	0.740
rs35656245	1	Intergenic	A	G	0.28	0.016	0.003	2.23E-08		0.27	-0.013	0.007	0.080
rs3820277	1	Intron:IGSF21	G	T	0.47	0.019	0.003	1.57E-13		0.47	0.003	0.006	0.650

rs45444697	1	Intron:ADAM15|DCST1-AS1	G	C	0.21	0.020	0.003	2.72E-10	0.22	0.009	0.008	0.270
rs4912332	1	Intergenic	T	C	0.49	0.014	0.003	2.94E-08	0.49	0.012	0.006	0.067
rs80054503	1	Intergenic	T	C	0.88	0.024	0.004	3.10E-09	0.99	0.045	0.055	0.410
rs876793	1	Intron:RYR2	T	C	0.65	0.018	0.003	5.69E-11	0.65	0.008	0.007	0.220
rs925524	1	Synonymous:MAST2	G	A	0.71	0.016	0.003	2.94E-08	0.72	0.025	0.007	0.001
rs951740	1	Intron:PTPRF	A	G	0.63	0.030	0.003	3.82E-29	0.62	0.005	0.007	0.440
rs1004787	2	Intron:LINC01833	A	G	0.55	0.028	0.003	1.11E-28	0.55	-0.012	0.006	0.065
rs1022376	2	Intergenic	T	C	0.48	0.015	0.003	1.66E-08	0.48	0.009	0.006	0.160
rs10490159	2	Intron:LOC730100	T	C	0.39	0.017	0.003	3.86E-11	0.40	-0.003	0.007	0.670
rs114976176	2	Intron:SH3YL1	A	C	0.65	0.016	0.003	6.04E-09	0.65	0.013	0.007	0.044
rs11678980	2	Exon:LINC01806	A	G	0.45	0.018	0.003	5.19E-12	0.45	0.017	0.007	0.008
rs11692435	2	Nonsynonymous:ACTR1B	A	G	0.08	0.025	0.005	4.47E-08	0.10	0.003	0.012	0.780
rs11889814	2	Intergenic	A	C	0.87	0.021	0.004	3.44E-08	0.87	0.011	0.009	0.260
rs12474587	2	Intron:SLC4A10	T	G	0.43	0.024	0.003	4.83E-21	0.43	-0.015	0.006	0.022
rs12714017	2	Intergenic	C	T	0.51	0.015	0.003	3.65E-09	0.51	0.007	0.006	0.240
rs13007361	2	Intergenic	A	G	0.21	0.018	0.003	2.29E-08	0.20	0.001	0.008	0.870
rs13392222	2	Intron:AFF3	A	C	0.86	0.023	0.004	1.93E-10	0.86	-0.002	0.009	0.820
rs1445649	2	Intron:KCNJ3	C	T	0.54	0.021	0.003	8.48E-16	0.53	-0.009	0.006	0.170
rs1518393	2	Intron:VRK2	C	A	0.62	0.017	0.003	1.30E-10	0.62	0.006	0.007	0.370
rs16826827	2	Intergenic	T	C	0.88	0.022	0.004	9.17E-09	0.88	-0.010	0.010	0.290
rs1722666	2	Intergenic	T	C	0.73	0.016	0.003	2.17E-08	0.73	-0.011	0.007	0.110
rs17229285	2	Intergenic	C	T	0.50	0.016	0.003	1.27E-09	0.50	0.000	0.006	0.990
rs17616642	2	Intron:LINC01122	A	G	0.75	0.017	0.003	2.10E-08	0.75	-0.011	0.007	0.150
rs1863161	2	Intergenic	A	G	0.56	0.015	0.003	2.34E-09	0.57	0.004	0.006	0.520
rs1901477	2	Intergenic	G	A	0.51	0.030	0.003	2.07E-31	0.51	0.026	0.007	0.000
rs2539706	2	Intergenic	A	G	0.53	0.016	0.003	1.95E-10	0.52	0.007	0.006	0.280
rs2710634	2	Intron:BIRC6	T	C	0.48	0.018	0.003	3.36E-12	0.48	0.010	0.006	0.120
rs3076896	2	Intergenic	A	G	0.39	0.023	0.003	1.99E-16	0.00	0.100	0.074	0.180
rs3115418	2	Intergenic	T	C	0.55	0.014	0.003	2.79E-08	0.55	0.014	0.006	0.031

rs34399632	2	Intron:THSD7B	G	A	0.23	0.019	0.003	1.46E-10	0.25	0.005	0.007	0.490
rs357304	2	Intergenic	C	T	0.73	0.017	0.003	5.40E-09	0.73	0.001	0.007	0.930
rs359247	2	Intergenic	T	A	0.64	0.022	0.003	9.89E-17	0.64	0.014	0.007	0.031
rs3811038	2	Intron:TTL	C	T	0.28	0.019	0.003	1.58E-11	0.29	0.014	0.007	0.052
rs4674916	2	Intron:CUL3	C	A	0.67	0.018	0.003	3.06E-11	0.68	0.003	0.007	0.660
rs4674993	2	Intron:NYAP2	A	G	0.80	0.024	0.003	4.85E-14	0.80	-0.014	0.008	0.074
rs56208390	2	Intergenic	G	A	0.12	0.022	0.004	2.68E-08	0.12	0.014	0.010	0.140
rs61533748	2	Intergenic	C	T	0.38	0.017	0.003	2.82E-11	0.39	0.027	0.007	0.000
rs62106258	2	Intergenic	T	C	0.95	0.046	0.006	3.33E-14	0.95	0.110	0.016	0.000
rs62137126	2	Intergenic	A	G	0.88	0.024	0.004	1.31E-09	0.88	-0.021	0.010	0.031
rs62180324	2	Intron:WDPCP	G	A	0.79	0.020	0.003	3.91E-10	0.79	-0.001	0.008	0.900
rs62193862	2	Intergenic	A	G	0.10	0.024	0.004	1.99E-08	0.10	-0.004	0.011	0.690
rs6730325	2	Intergenic	G	A	0.39	0.015	0.003	2.10E-08	0.39	0.036	0.007	0.000
rs6731872	2	Intergenic	G	T	0.83	0.032	0.003	5.35E-21	0.83	0.053	0.009	0.000
rs6750107	2	Intron:CTNNA2	A	G	0.39	0.015	0.003	2.60E-08	0.40	-0.019	0.007	0.003
rs6750529	2	Intron:LINC01934	T	C	0.74	0.020	0.003	9.26E-12	0.74	0.004	0.007	0.620
rs6756212	2	Intergenic	C	T	0.46	0.034	0.003	3.49E-40	0.47	-0.011	0.006	0.077
rs72790288	2	Intron:ALK	G	A	0.97	0.046	0.008	3.28E-09	0.97	-0.010	0.019	0.600
rs74697736	2	Intergenic	A	G	0.29	0.022	0.003	2.43E-15	0.29	-0.003	0.007	0.700
rs75210106	2	Intron:TTL	C	T	0.82	0.019	0.003	2.33E-08	0.82	0.012	0.008	0.160
rs7585579	2	Intergenic	G	C	0.50	0.020	0.003	5.48E-15	0.50	0.006	0.007	0.400
rs7598402	2	Intron:NRXN1	C	G	0.51	0.015	0.003	7.38E-09	0.51	-0.002	0.006	0.800
rs7600835	2	Intergenic	G	A	0.66	0.015	0.003	1.80E-08	0.65	0.005	0.007	0.500
rs10446419	3	Intergenic	A	G	0.79	0.020	0.003	5.05E-10	0.79	0.011	0.008	0.160
rs10935779	3	Intron:RNF13	C	T	0.58	0.014	0.003	2.95E-08	0.58	0.012	0.006	0.065
rs11128203	3	Intron:FOXP1	A	T	0.53	0.020	0.003	1.29E-15	0.53	0.001	0.006	0.840
rs11713899	3	Intron:CNTN4	C	A	0.17	0.019	0.003	3.15E-08	0.17	0.006	0.009	0.510
rs1187820	3	Intergenic	C	T	0.56	0.014	0.003	2.69E-08	0.56	0.020	0.007	0.003
rs12053870	3	Intron:LOC105374060	G	T	0.54	0.016	0.003	1.02E-09	0.54	0.005	0.006	0.420

rs12633090	3	Intergenic	G	C	0.82	0.023	0.003	3.16E-12	0.82	0.006	0.008	0.450
rs13066050	3	Intergenic	T	C	0.21	0.019	0.003	1.93E-09	0.21	0.004	0.008	0.630
rs13319205	3	Intron:SMARCC1	A	T	0.29	0.017	0.003	3.77E-09	0.29	-0.020	0.007	0.005
rs1449012	3	Intron:IQCJ-SCHIP1|SCHIP1	C	T	0.54	0.015	0.003	1.77E-09	0.53	-0.003	0.006	0.700
rs1549979	3	Intron:CADM2	C	T	0.39	0.025	0.003	8.80E-21	0.38	0.001	0.007	0.900
rs16828799	3	Intron:NLGN1	T	G	0.16	0.020	0.004	1.83E-08	0.16	-0.013	0.009	0.130
rs1714521	3	Intron:LOC100996447	A	C	0.59	0.016	0.003	3.07E-10	0.58	-0.016	0.006	0.012
rs1910236	3	Intergenic	A	G	0.47	0.015	0.003	9.91E-09	0.47	-0.005	0.006	0.460
rs2196356	3	Intergenic	G	C	0.71	0.019	0.003	2.45E-11	0.71	0.007	0.007	0.340
rs221988	3	Intergenic	A	C	0.62	0.015	0.003	1.43E-08	0.61	0.003	0.007	0.610
rs2276825	3	Intron:STIMATE|TMEM110-MUSTN1	C	T	0.25	0.019	0.003	1.89E-10	0.25	0.024	0.007	0.001
rs2279829	3	Utr3:ZIC4	C	T	0.78	0.017	0.003	2.05E-08	0.78	-0.013	0.008	0.084
rs2306866	3	Intron:CACNA1D	A	T	0.39	0.017	0.003	1.89E-10	0.38	-0.002	0.007	0.750
rs2319545	3	Intergenic	A	C	0.15	0.023	0.004	8.30E-11	0.15	-0.003	0.009	0.740
rs2526390	3	Intron:SEMA3F|SEMA3F-AS1	T	C	0.33	0.021	0.003	3.62E-14	0.33	0.010	0.007	0.160
rs2734390	3	Intron:FHIT	G	A	0.37	0.015	0.003	2.09E-08	0.37	0.010	0.007	0.150
rs3172494	3	Utr3:IP6K2	G	T	0.89	0.029	0.004	3.40E-13	0.88	0.013	0.010	0.220
rs4543050	3	Intergenic	T	A	0.82	0.022	0.003	1.45E-11	0.82	0.015	0.008	0.064
rs57153235	3	Intron:CADM2	T	G	0.68	0.019	0.003	1.56E-12	0.68	0.015	0.007	0.024
rs62246017	3	Intron:FOXP1	G	A	0.68	0.016	0.003	3.03E-09	0.68	0.013	0.007	0.070
rs6437769	3	Intergenic	T	C	0.58	0.014	0.003	3.74E-08	0.58	0.013	0.007	0.039
rs6438436	3	Intergenic	T	C	0.82	0.025	0.003	5.33E-14	0.81	0.012	0.008	0.150
rs6782116	3	Intron:ROBO2	C	T	0.58	0.015	0.003	1.46E-08	0.58	-0.013	0.007	0.054
rs73831818	3	Intron:ERC2	G	A	0.06	0.032	0.006	5.46E-09	0.06	0.026	0.014	0.056
rs74664784	3	Intron:CADM2	T	C	0.62	0.020	0.003	9.34E-13	1.00	0.120	0.190	0.520
rs748832	3	Intergenic	G	A	0.37	0.017	0.003	6.60E-11	0.37	0.012	0.007	0.081
rs7631379	3	Intron:SOX2-OT	C	T	0.21	0.021	0.003	3.94E-11	0.20	0.015	0.008	0.061
rs7640107	3	Intron:FHIT	C	T	0.57	0.014	0.003	3.46E-08	0.57	-0.002	0.006	0.720

rs9288999	3	Intron:ZBTB20	A	G	0.74	0.017	0.003	1.50E-09	0.73	0.000	0.007	1.000
rs963354	3	Intergenic	A	C	0.69	0.015	0.003	4.21E-08	0.68	-0.013	0.007	0.061
rs9826984	3	Intergenic	G	A	0.46	0.014	0.003	3.87E-08	0.46	-0.005	0.006	0.460
rs9841807	3	Intergenic	T	C	0.27	0.016	0.003	1.35E-08	0.28	0.002	0.007	0.780
rs9850597	3	Intergenic	G	A	0.18	0.019	0.003	1.65E-08	0.18	0.004	0.008	0.590
rs1116690	4	Intron:INPP4B	G	A	0.74	0.016	0.003	2.16E-08	0.75	-0.025	0.007	0.001
rs112725451	4	Intergenic	T	C	0.17	0.026	0.003	1.65E-14	0.17	0.012	0.009	0.160
rs1160685	4	Intron:GRID2	G	C	0.45	0.015	0.003	2.31E-09	0.44	-0.003	0.006	0.690
rs12642744	4	Intergenic	G	T	0.26	0.017	0.003	2.82E-08	0.25	0.013	0.008	0.090
rs13109980	4	Intron:MAML3	G	A	0.67	0.022	0.003	3.37E-16	0.67	0.031	0.007	0.000
rs13110073	4	Intron:TTC29	T	C	0.61	0.025	0.003	3.24E-21	0.60	-0.003	0.007	0.660
rs1389171	4	Intergenic	T	A	0.76	0.018	0.003	4.45E-09	0.76	0.019	0.007	0.009
rs1435479	4	Intron:GRID2	T	G	0.29	0.016	0.003	5.68E-09	0.29	0.023	0.007	0.001
rs28717373	4	Intergenic	C	T	0.64	0.017	0.003	6.16E-10	0.63	-0.004	0.007	0.570
rs3934797	4	Intergenic	G	A	0.82	0.021	0.003	1.12E-10	0.82	-0.002	0.008	0.810
rs4140932	4	Intergenic	T	A	0.57	0.014	0.003	4.89E-08	0.57	0.004	0.006	0.590
rs55900829	4	Intergenic	T	A	0.33	0.019	0.003	5.63E-12	0.44	0.021	0.018	0.240
rs55944129	4	Intergenic	T	C	0.73	0.018	0.003	1.06E-09	0.72	0.006	0.007	0.410
rs58400863	4	Intron:LINC02497	G	A	0.65	0.020	0.003	4.89E-14	0.65	-0.002	0.007	0.790
rs59537158	4	Intergenic	T	C	0.21	0.023	0.003	4.62E-13	0.21	0.007	0.008	0.390
rs62340589	4	Intron:GPM6A	C	G	0.20	0.017	0.003	4.31E-08	0.21	0.001	0.008	0.910
rs71602617	4	Intergenic	C	T	0.78	0.018	0.003	2.10E-08	0.78	-0.006	0.008	0.420
rs7657022	4	Intergenic	G	A	0.49	0.018	0.003	7.34E-13	0.49	0.007	0.006	0.310
rs7696257	4	Intergenic	A	G	0.37	0.015	0.003	6.78E-09	0.37	0.000	0.007	0.960
rs10042827	5	Intron:RANBP17	C	T	0.68	0.017	0.003	9.41E-10	0.68	0.013	0.007	0.054
rs10060196	5	Intergenic	A	C	0.58	0.018	0.003	1.29E-12	0.58	-0.006	0.006	0.320
rs10805858	5	Intergenic	T	A	0.34	0.018	0.003	1.88E-11	0.34	0.016	0.007	0.018
rs1173461	5	Intergenic	T	C	0.33	0.017	0.003	9.51E-10	0.33	0.001	0.007	0.920
rs11956866	5	Intergenic	T	G	0.43	0.015	0.003	7.82E-09	0.43	-0.006	0.006	0.360

rs12517438	5	Intergenic	G	T	0.54	0.015	0.003	1.89E-09	0.54	-0.003	0.006	0.640
rs1385108	5	Intergenic	T	C	0.24	0.019	0.003	3.84E-10	0.24	-0.003	0.007	0.700
rs17165769	5	Intron:FBXL17	G	A	0.39	0.016	0.003	9.56E-10	0.39	0.004	0.006	0.520
rs181508347	5	Intergenic	G	T	0.01	0.081	0.013	4.95E-10	0.01	0.020	0.033	0.550
rs2028269	5	Intron:THBS4	A	G	0.40	0.016	0.003	5.19E-10	0.40	0.016	0.006	0.014
rs2173019	5	Intron:TENM2	A	T	0.18	0.028	0.003	2.98E-17	0.17	-0.001	0.009	0.950
rs329124	5	Intron:JADE2	A	G	0.57	0.016	0.003	1.96E-10	0.57	-0.036	0.006	0.000
rs35375873	5	Intergenic	G	C	0.89	0.027	0.004	3.29E-11	0.88	0.030	0.010	0.004
rs359431	5	Intergenic	C	T	0.44	0.014	0.003	3.16E-08	0.45	0.006	0.006	0.340
rs3843905	5	Intergenic	C	T	0.60	0.015	0.003	5.41E-09	0.60	0.007	0.007	0.270
rs3909281	5	Intergenic	G	T	0.54	0.021	0.003	1.62E-16	0.53	0.019	0.006	0.003
rs4044321	5	Intron:TENM2	A	G	0.36	0.023	0.003	1.75E-17	0.36	0.007	0.007	0.320
rs42417	5	Intron:MCTP1	T	C	0.69	0.017	0.003	8.27E-10	0.68	0.012	0.007	0.092
rs6452785	5	Intron:TMEM161B-AS1	C	T	0.53	0.027	0.003	4.69E-26	0.53	0.019	0.006	0.002
rs6874731	5	Intron:RASGRF2	G	T	0.48	0.015	0.003	1.83E-09	0.49	-0.002	0.006	0.780
rs6890961	5	Intron:TENM2	C	T	0.38	0.019	0.003	2.13E-13	0.38	-0.001	0.007	0.870
rs71592686	5	Intron:ELOVL7	C	T	0.27	0.021	0.003	3.85E-13	0.28	0.005	0.007	0.520
rs72780746	5	Intergenic	T	C	0.83	0.026	0.003	2.05E-14	0.82	0.014	0.009	0.110
rs72789626	5	Intron:EFNA5	T	A	0.86	0.026	0.004	5.13E-12	0.87	0.008	0.010	0.380
rs79476395	5	Intergenic	G	A	0.07	0.033	0.005	1.04E-11	0.07	-0.004	0.013	0.780
rs986714	5	Intergenic	A	T	0.55	0.016	0.003	4.13E-10	0.55	0.004	0.006	0.540
rs1059490	6	Utr3:HIST1H2BD	T	C	0.63	0.019	0.003	2.16E-12	0.63	0.006	0.007	0.370
rs10698713	6	Intron:TULP4	G	A	0.95	0.034	0.006	2.38E-09	0.95	-0.014	0.014	0.320
rs10945141	6	Intron:ADGRB3	A	G	0.26	0.018	0.003	3.59E-10	0.26	0.012	0.007	0.100
rs1150668	6	Intron:ZNF192P1	T	G	0.58	0.019	0.003	8.54E-13	0.58	0.007	0.007	0.270
rs118202	6	Intron:REV3L	G	T	0.19	0.037	0.003	1.90E-29	0.19	0.025	0.008	0.002
rs12195240	6	Intergenic	A	G	0.28	0.025	0.003	1.08E-18	0.29	-0.007	0.007	0.290
rs12530388	6	Utr5:ASCC3	A	C	0.49	0.018	0.003	5.83E-13	0.49	0.003	0.006	0.610
rs160631	6	Intron:ICK	T	G	0.27	0.017	0.003	1.87E-09	0.27	0.009	0.007	0.210

rs1632941	6	Intron:HLA-G	T	C	0.54	0.016	0.003	6.67E-10	0.54	0.000	0.007	0.990
rs1737329	6	Intergenic	G	C	0.74	0.017	0.003	5.08E-09	0.74	-0.023	0.007	0.001
rs17554906	6	Intergenic	C	G	0.44	0.014	0.003	3.14E-08	0.45	0.001	0.007	0.910
rs3218116	6	Intergenic	C	T	0.74	0.020	0.003	1.05E-11	0.75	0.006	0.007	0.450
rs3800227	6	Intron:FOXO3	G	A	0.74	0.017	0.003	3.64E-09	0.74	0.014	0.007	0.052
rs619087	6	Intergenic	G	A	0.42	0.014	0.003	3.10E-08	0.43	0.003	0.006	0.700
rs6568832	6	Intron:MIR548H3|MMS22L	A	G	0.75	0.019	0.003	1.74E-10	0.75	0.012	0.007	0.110
rs6932350	6	Intron:LOC105374988	A	T	0.45	0.015	0.003	5.13E-09	0.46	0.005	0.006	0.460
rs6936160	6	Intergenic	T	C	0.70	0.020	0.003	4.20E-13	0.70	-0.014	0.007	0.044
rs73008357	6	Intergenic	A	C	0.88	0.022	0.004	2.44E-08	0.88	-0.003	0.010	0.750
rs7743165	6	Intergenic	G	T	0.50	0.019	0.003	4.15E-14	0.49	0.012	0.006	0.051
rs79180767	6	Intergenic	T	C	0.25	0.020	0.003	7.00E-12	0.32	0.012	0.020	0.550
rs9331343	6	Intron:TMEM242	T	C	0.43	0.014	0.003	3.90E-08	0.43	0.002	0.007	0.720
rs10233018	7	Intergenic	G	A	0.52	0.025	0.003	4.77E-22	0.52	0.022	0.006	0.001
rs10259715	7	Intergenic	T	A	0.79	0.019	0.003	6.42E-09	0.74	0.013	0.021	0.530
rs10272990	7	Intergenic	T	C	0.67	0.021	0.003	1.27E-14	0.66	-0.005	0.007	0.470
rs10279261	7	Intron:EXOC4	G	A	0.38	0.019	0.003	6.05E-13	0.39	0.010	0.007	0.130
rs1030015	7	Intron:MAGI2	T	G	0.52	0.014	0.003	2.15E-08	0.51	0.021	0.006	0.001
rs10953957	7	Intergenic	A	G	0.39	0.014	0.003	3.66E-08	0.39	0.026	0.007	0.000
rs112913817	7	Intergenic	G	A	0.01	0.078	0.012	9.28E-11	0.01	0.008	0.032	0.810
rs11766326	7	Intron:IMMP2L	T	C	0.49	0.018	0.003	1.79E-11	0.48	0.003	0.006	0.690
rs11768481	7	Intron:DLX6-AS1	C	A	0.66	0.019	0.003	5.23E-12	0.65	0.002	0.007	0.810
rs13237637	7	Intron:SDK1	G	C	0.52	0.024	0.003	1.54E-20	0.51	0.004	0.006	0.580
rs13437771	7	Intron:ZNF789	A	G	0.85	0.027	0.004	1.39E-14	0.84	0.028	0.009	0.002
rs1561112	7	Intron:LRGUK	T	C	0.59	0.015	0.003	3.84E-09	0.59	-0.001	0.007	0.930
rs1799068	7	Intergenic	T	G	0.38	0.017	0.003	2.59E-10	0.38	-0.012	0.007	0.058
rs4727189	7	Intron:ZNF804B	C	T	0.34	0.015	0.003	3.00E-08	0.34	-0.005	0.007	0.480
rs6948707	7	Intron:MAD1L1	G	T	0.42	0.024	0.003	4.24E-21	0.42	0.008	0.007	0.220
rs6968380	7	Intergenic	G	A	0.32	0.023	0.003	1.05E-17	0.32	0.015	0.007	0.028

rs76841737	7	Intergenic	C	G	0.90	0.023	0.004	3.26E-08	0.90	0.006	0.011	0.550
rs77283305	7	Intron:CHCHD3	G	A	0.69	0.015	0.003	3.91E-08	0.70	0.019	0.007	0.006
rs7802996	7	Intron:MAGI2	C	T	0.83	0.021	0.003	1.06E-09	0.83	-0.006	0.009	0.520
rs7809303	7	Intron:AUTS2	G	A	0.68	0.021	0.003	3.48E-15	0.67	-0.023	0.007	0.001
rs79631993	7	Intron:AUTS2	A	C	0.78	0.017	0.003	3.67E-08	0.66	-0.017	0.019	0.380
rs11780471	8	Intergenic	G	A	0.94	0.039	0.005	1.57E-13	0.94	-0.025	0.013	0.057
rs11783093	8	Intergenic	C	T	0.84	0.047	0.004	2.07E-41	0.84	-0.009	0.009	0.320
rs13261666	8	Intron:TOX	G	T	0.48	0.020	0.003	4.36E-15	0.49	0.015	0.006	0.020
rs1565735	8	Intergenic	T	A	0.80	0.019	0.003	1.33E-09	0.80	-0.006	0.008	0.470
rs2063976	8	Intergenic	C	T	0.34	0.020	0.003	7.45E-14	0.34	-0.017	0.007	0.014
rs290601	8	Intergenic	T	C	0.27	0.016	0.003	1.14E-08	0.28	0.018	0.007	0.012
rs2952251	8	Intron:MSRA	G	A	0.74	0.016	0.003	4.24E-08	0.74	0.018	0.008	0.025
rs3850736	8	Intron:LOC102724623	G	C	0.47	0.019	0.003	6.43E-14	0.47	-0.022	0.006	0.001
rs4326350	8	Intron:XKR6	C	G	0.51	0.018	0.003	5.16E-12	0.51	0.042	0.006	0.000
rs6986430	8	Intron:RUNX1T1	T	C	0.78	0.024	0.003	1.99E-15	0.78	0.013	0.008	0.100
rs6993429	8	Intergenic	C	A	0.55	0.019	0.003	9.87E-14	0.55	0.006	0.006	0.340
rs7836565	8	Intron:PXDNL	C	T	0.28	0.016	0.003	4.36E-08	0.28	-0.006	0.007	0.360
rs9987376	8	Intergenic	T	G	0.43	0.021	0.003	2.01E-15	0.42	0.009	0.006	0.150
rs10858334	9	Utr3:OLFM1	G	C	0.14	0.023	0.004	1.18E-09	0.14	0.033	0.010	0.001
rs10966092	9	Intron:ELAVL2	T	C	0.73	0.021	0.003	1.12E-12	0.73	0.013	0.007	0.061
rs10969352	9	Intergenic	A	T	0.50	0.014	0.003	1.82E-08	0.49	0.016	0.006	0.011
rs11791671	9	Intron:RFX3	T	C	0.07	0.028	0.005	4.24E-08	0.07	0.009	0.013	0.470
rs1759433	9	Intron:GAPVD1	A	G	0.48	0.015	0.003	1.69E-09	0.48	-0.022	0.006	0.001
rs1927901	9	Intergenic	T	C	0.45	0.014	0.003	3.10E-08	0.45	0.011	0.006	0.091
rs1930371	9	Intergenic	C	T	0.76	0.017	0.003	7.09E-09	0.76	-0.010	0.007	0.170
rs1931431	9	Intergenic	C	G	0.48	0.018	0.003	8.56E-13	0.48	0.006	0.006	0.340
rs2378662	9	Intron:LOC101927575	A	G	0.54	0.015	0.003	2.67E-09	0.54	0.007	0.006	0.260
rs34553878	9	Nonsynonymous:PRRC2B	G	A	0.11	0.025	0.004	1.17E-09	0.11	-0.011	0.010	0.260
rs3847244	9	Intergenic	T	C	0.47	0.019	0.003	2.60E-13	0.46	-0.002	0.006	0.780

rs4837631	9	Intron:BRINP1	C	T	0.55	0.015	0.003	2.03E-09	0.55	0.011	0.006	0.094
rs4877285	9	Intergenic	G	A	0.33	0.018	0.003	2.10E-11	0.33	0.029	0.007	0.000
rs6474609	9	Intergenic	T	A	0.41	0.016	0.003	1.71E-09	0.41	0.010	0.006	0.140
rs7024924	9	Intergenic	C	T	0.17	0.019	0.003	1.90E-08	0.18	0.028	0.008	0.001
rs7026534	9	Intron:MED27	T	G	0.30	0.017	0.003	2.68E-09	0.30	0.024	0.007	0.001
rs7867822	9	Intron:FOCAD	A	G	0.33	0.015	0.003	2.76E-08	0.33	-0.025	0.007	0.000
rs10885480	10	Intron:NRAP	T	C	0.72	0.019	0.003	3.83E-11	0.71	0.001	0.007	0.900
rs10905461	10	Intergenic	T	C	0.25	0.016	0.003	2.36E-08	0.24	-0.001	0.007	0.910
rs11191269	10	Intron:GBF1	G	C	0.19	0.018	0.003	4.61E-08	0.20	-0.010	0.008	0.220
rs11192347	10	Intron:SORCS3	G	A	0.90	0.027	0.004	6.15E-10	0.89	-0.005	0.011	0.620
rs11258417	10	Intron:BEND7	C	T	0.61	0.015	0.003	2.71E-08	0.61	0.024	0.007	0.000
rs11594623	10	Intergenic	C	T	0.23	0.027	0.003	7.45E-20	0.24	0.006	0.008	0.410
rs12244388	10	Intron:AS3MT|BORCS7-ASMT	A	G	0.35	0.026	0.003	4.31E-22	0.35	0.003	0.007	0.650
rs1291821	10	Intron:CELF2|CELF2-AS2	G	A	0.53	0.015	0.003	1.39E-08	0.53	0.015	0.006	0.018
rs1733760	10	Intron:PCDH15	C	T	0.51	0.015	0.003	6.70E-09	0.51	0.011	0.006	0.086
rs2796793	10	Intergenic	A	G	0.45	0.015	0.003	1.55E-08	0.46	0.009	0.006	0.150
rs28408682	10	Intergenic	G	A	0.60	0.017	0.003	1.41E-10	0.59	0.017	0.007	0.010
rs34970111	10	Intron:ITPRIP	C	T	0.54	0.015	0.003	1.28E-08	0.54	0.004	0.006	0.580
rs4752018	10	Intron:SHTN1	A	C	0.23	0.019	0.003	4.42E-10	0.23	0.020	0.008	0.007
rs7072776	10	Intergenic	A	G	0.29	0.022	0.003	5.66E-15	0.28	0.006	0.007	0.390
rs7901883	10	Intron:BTRC	G	A	0.77	0.019	0.003	1.98E-10	0.77	-0.009	0.007	0.250
rs7920501	10	Intergenic	T	A	0.53	0.016	0.003	1.25E-09	0.54	0.002	0.006	0.800
rs7921378	10	Intron:ARID5B	G	C	0.52	0.023	0.003	6.10E-20	0.52	-0.015	0.007	0.021
rs9423279	10	Intergenic	C	G	0.36	0.019	0.003	3.06E-12	0.35	-0.005	0.007	0.450
rs9787523	10	Intron:SORCS3	T	C	0.58	0.016	0.003	1.42E-09	0.58	0.018	0.007	0.005
rs1106363	11	Intron:NTM	T	C	0.34	0.017	0.003	9.20E-11	0.35	0.011	0.007	0.110
rs1381775	11	Intergenic	T	C	0.29	0.016	0.003	2.79E-08	0.28	-0.012	0.007	0.099
rs1713676	11	Intergenic	A	G	0.48	0.017	0.003	5.38E-11	0.48	0.004	0.006	0.500
rs1834306	11	Intron:MIR100HG	A	G	0.42	0.015	0.003	1.96E-08	0.42	0.024	0.007	0.000

rs1944689	11	Intergenic	T	G	0.79	0.018	0.003	1.27E-08	0.79	-0.003	0.008	0.700
rs2010921	11	Intron:NTM	A	G	0.31	0.017	0.003	2.47E-10	0.31	0.003	0.007	0.620
rs2155646	11	Intron:NCAM1	C	T	0.40	0.038	0.003	9.44E-48	0.40	-0.010	0.007	0.120
rs238896	11	Intron:ZBTB16	G	A	0.51	0.017	0.003	3.65E-11	0.51	-0.005	0.006	0.470
rs2939756	11	Intron:LRRC4C	G	A	0.52	0.016	0.003	7.45E-10	0.53	0.005	0.006	0.400
rs2959084	11	Intron:PHF21A	A	G	0.70	0.017	0.003	9.82E-10	0.70	-0.010	0.007	0.130
rs3740977	11	Intron:DGKZ	C	T	0.17	0.020	0.003	1.17E-08	0.17	0.004	0.009	0.620
rs4275621	11	Intergenic	A	G	0.62	0.021	0.003	3.76E-16	0.62	0.007	0.007	0.280
rs540860	11	Intergenic	G	A	0.54	0.018	0.003	5.75E-12	0.54	-0.013	0.006	0.047
rs586699	11	Intron:FAT3	G	A	0.46	0.015	0.003	7.29E-09	0.46	0.005	0.006	0.410
rs61884449	11	Intron:NRXN2	T	C	0.15	0.020	0.004	2.32E-08	0.15	-0.001	0.009	0.940
rs61886926	11	Intron:RPS6KA4	C	T	0.62	0.018	0.003	7.30E-12	0.61	0.010	0.007	0.110
rs62618693	11	Nonsynonymous:QSER1	C	T	0.96	0.035	0.006	2.09E-08	0.96	0.110	0.016	0.000
rs6265	11	Nonsynonymous:BDNF	C	T	0.81	0.029	0.003	2.81E-19	0.81	0.016	0.008	0.054
rs644740	11	Intron:OVOL1	C	T	0.54	0.014	0.003	3.67E-08	0.54	0.017	0.006	0.008
rs76460663	11	Intergenic	C	G	0.96	0.042	0.006	4.15E-11	0.95	-0.001	0.016	0.960
rs78239456	11	Intron:NCAM1	A	T	0.62	0.019	0.003	9.37E-12	0.53	-0.012	0.020	0.550
rs7929518	11	Intron:EED	G	A	0.77	0.019	0.003	2.55E-10	0.77	0.009	0.008	0.250
rs7943721	11	Intergenic	G	A	0.17	0.021	0.003	3.58E-10	0.17	0.007	0.009	0.420
rs11057005	12	Intron:LMO3	A	G	0.56	0.016	0.003	9.12E-10	0.55	0.007	0.006	0.290
rs1109480	12	Intron:CABP1	G	A	0.62	0.017	0.003	1.84E-10	0.61	0.034	0.007	0.000
rs11611651	12	Intron:GOLGA3	A	G	0.09	0.027	0.005	2.05E-09	0.09	0.027	0.011	0.018
rs13906	12	Utr3:MCRS1	C	T	0.89	0.025	0.004	1.98E-09	0.89	0.029	0.010	0.005
rs4759229	12	Intron:ERBB3	G	A	0.66	0.016	0.003	6.53E-09	0.66	0.006	0.007	0.360
rs7134009	12	Intergenic	T	C	0.71	0.016	0.003	4.30E-08	0.71	-0.004	0.007	0.570
rs77215829	12	Intron:HECTD4	A	C	0.87	0.024	0.004	2.02E-10	0.86	0.025	0.010	0.007
rs7969559	12	Intron:CPSF6	A	G	0.29	0.017	0.003	1.53E-09	0.29	0.022	0.007	0.002
rs1108130	13	Exon:LINC00554	A	T	0.21	0.024	0.003	1.57E-14	0.22	0.003	0.008	0.740
rs12855717	13	Intergenic	T	C	0.54	0.016	0.003	1.22E-09	0.54	0.005	0.006	0.480

rs1413119	13	Intergenic	C	T	0.60	0.015	0.003	4.77E-09	0.60	0.021	0.007	0.002
rs17197663	13	Utr5:POSTN	G	A	0.88	0.022	0.004	2.06E-08	0.87	0.006	0.010	0.540
rs1772572	13	Intergenic	C	A	0.68	0.017	0.003	5.62E-10	0.67	-0.002	0.007	0.770
rs3098272	13	Intergenic	A	C	0.20	0.018	0.003	2.08E-08	0.20	-0.013	0.008	0.100
rs4264267	13	Intron:TRPC4	T	C	0.53	0.015	0.003	6.82E-09	0.53	0.008	0.006	0.240
rs4886207	13	Intron:DIAPH3	T	C	0.36	0.016	0.003	8.78E-10	0.36	0.013	0.007	0.050
rs55786907	13	Intergenic	G	A	0.16	0.019	0.004	1.84E-08	0.16	0.017	0.009	0.048
rs56367474	13	Intergenic	C	T	0.70	0.017	0.003	4.20E-10	0.70	-0.017	0.007	0.015
rs61959481	13	Intergenic	G	A	0.79	0.020	0.003	7.95E-11	0.79	-0.006	0.008	0.430
rs7333559	13	Intron:CLYBL|LOC101927437	G	A	0.22	0.023	0.003	5.94E-14	0.21	0.017	0.008	0.031
rs75674569	13	Intron:HS6ST3	G	A	0.90	0.025	0.004	2.58E-09	0.90	-0.013	0.011	0.240
rs9538162	13	Intergenic	C	T	0.42	0.017	0.003	1.76E-11	0.42	-0.022	0.006	0.001
rs9540731	13	Intron:PCDH9	C	T	0.49	0.018	0.003	3.42E-12	0.49	0.009	0.006	0.140
rs9545155	13	Intergenic	T	C	0.52	0.016	0.003	3.04E-10	0.53	0.013	0.006	0.044
rs12878369	14	Intergenic	A	C	0.41	0.017	0.003	1.60E-11	0.41	-0.002	0.007	0.710
rs1381287	14	Intergenic	T	C	0.47	0.018	0.003	1.81E-12	0.46	0.011	0.006	0.082
rs1811739	14	Intron:LINC02288	A	G	0.25	0.018	0.003	5.97E-10	0.25	0.009	0.007	0.220
rs2145451	14	Intergenic	T	C	0.81	0.020	0.003	5.44E-10	0.81	-0.001	0.008	0.940
rs2925128	14	Intergenic	T	C	0.39	0.017	0.003	3.67E-10	0.39	-0.007	0.007	0.320
rs34940743	14	Intron:NRXN3	G	A	0.35	0.016	0.003	2.80E-09	0.34	0.009	0.007	0.200
rs55913542	14	Intron:BCL11B	T	G	0.18	0.019	0.003	3.25E-08	0.18	0.015	0.008	0.071
rs8005334	14	Intron:NRXN3	G	T	0.36	0.017	0.003	3.44E-10	0.36	-0.022	0.007	0.001
rs9323328	14	Intergenic	A	G	0.46	0.014	0.003	2.55E-08	0.46	0.024	0.006	0.000
rs12442563	15	Intergenic	G	T	0.78	0.023	0.003	3.13E-14	0.77	0.010	0.008	0.200
rs1435672	15	Intergenic	C	T	0.56	0.014	0.003	3.82E-08	0.56	0.016	0.006	0.012
rs1435741	15	Intron:SEMA6D	A	G	0.43	0.018	0.003	1.09E-12	0.43	-0.002	0.006	0.720
rs2289791	15	Intron:SMAD3	G	T	0.75	0.018	0.003	2.01E-09	0.75	0.010	0.007	0.180
rs281296	15	Intron:SEMA6D	A	G	0.36	0.025	0.003	1.59E-20	0.35	0.003	0.007	0.630
rs4310804	15	Intron:NR2F2-AS1	C	G	0.75	0.018	0.003	7.55E-10	0.75	0.010	0.007	0.160

rs56902655	15	Intergenic	T	G	0.86	0.022	0.004	4.09E-09	0.86	-0.010	0.009	0.300
rs60833441	15	Intergenic	A	G	0.54	0.014	0.003	2.28E-08	0.54	0.002	0.006	0.790
rs62007780	15	Intron:LINGO1	G	T	0.58	0.016	0.003	7.48E-10	0.59	0.001	0.007	0.940
rs8027457	15	Intron:IGF1R	C	T	0.51	0.015	0.003	1.88E-09	0.51	0.011	0.006	0.080
rs1050847	16	Utr3:ZCCHC14	C	T	0.44	0.015	0.003	7.37E-09	0.43	-0.007	0.006	0.310
rs11076962	16	Intergenic	C	T	0.28	0.018	0.003	1.20E-10	0.28	0.008	0.007	0.260
rs1139897	16	Nonsynonymous:RHOT2	G	A	0.77	0.024	0.003	1.77E-15	0.76	0.004	0.008	0.580
rs11642231	16	Intron:SPG7	G	A	0.63	0.016	0.003	3.44E-09	0.62	0.013	0.007	0.047
rs117657830	16	Intergenic	A	G	0.96	0.038	0.006	3.18E-09	0.96	-0.015	0.016	0.350
rs12918191	16	Intergenic	A	G	0.76	0.020	0.003	3.14E-11	0.76	-0.013	0.007	0.091
rs4785187	16	Intron:ZNF423	A	G	0.22	0.020	0.003	6.55E-11	0.23	-0.006	0.008	0.460
rs4788676	16	Intron:ZFHX3	T	C	0.77	0.018	0.003	4.92E-09	0.77	-0.010	0.008	0.190
rs61537885	16	Intergenic	T	C	0.96	0.040	0.007	8.06E-09	0.96	-0.004	0.046	0.930
rs62052916	16	Intron:LINC01572	A	T	0.93	0.032	0.005	1.62E-10	0.92	0.012	0.012	0.330
rs6497840	16	Intergenic	A	G	0.71	0.023	0.003	2.01E-15	0.71	-0.002	0.007	0.790
rs7188873	16	Intron:TNRC6A	G	A	0.61	0.020	0.003	8.46E-15	0.61	0.024	0.007	0.000
rs7192140	16	Intron:GRIN2A	T	C	0.50	0.017	0.003	3.40E-11	0.50	-0.006	0.006	0.360
rs8050598	16	Intergenic	T	C	0.25	0.019	0.003	1.76E-10	0.26	-0.006	0.008	0.400
rs9302604	16	Intergenic	G	A	0.44	0.019	0.003	3.29E-13	0.43	0.020	0.006	0.002
rs9922607	16	Intergenic	C	T	0.80	0.022	0.003	3.42E-12	0.80	0.008	0.008	0.310
rs9936784	16	Intergenic	G	T	0.53	0.014	0.003	4.33E-08	0.53	0.018	0.006	0.005
rs9941217	16	Intergenic	C	G	0.65	0.019	0.003	3.50E-12	0.64	0.002	0.007	0.810
rs11078713	17	Intron:CHD3	A	G	0.58	0.015	0.003	1.59E-08	0.58	-0.004	0.007	0.530
rs11651955	17	Intergenic	G	A	0.50	0.014	0.003	3.74E-08	0.51	-0.004	0.006	0.540
rs17692129	17	Intron:NSF	T	C	0.33	0.020	0.003	4.57E-13	0.34	0.017	0.007	0.017
rs2344976	17	Intron:ZNF207	T	C	0.39	0.015	0.003	7.98E-09	0.38	-0.005	0.007	0.450
rs2587507	17	Intergenic	T	C	0.50	0.015	0.003	8.69E-09	0.49	-0.004	0.006	0.490
rs28441558	17	Intron:CHD3	T	C	0.94	0.036	0.006	1.24E-10	0.94	-0.014	0.014	0.300
rs2938134	17	Intergenic	C	A	0.33	0.018	0.003	3.14E-10	0.33	0.004	0.007	0.540

rs3764351	17	Intron:PNMT	G	A	0.34	0.015	0.003	3.89E-08	0.34	0.027	0.007	0.000
rs4790874	17	Intron:SMG6	T	C	0.53	0.017	0.003	8.43E-12	0.53	-0.020	0.006	0.002
rs67777803	17	Intron:SEZ6	G	T	0.83	0.025	0.003	3.18E-13	0.83	0.004	0.008	0.650
rs72836318	17	Intron:KANSL1	T	C	0.75	0.017	0.003	7.00E-09	0.75	0.023	0.007	0.002
rs75919030	17	Intron:CA10	T	C	0.73	0.021	0.003	3.35E-13	0.73	-0.004	0.007	0.620
rs11872397	18	Intron:ZNF407	G	A	0.75	0.017	0.003	5.20E-09	0.75	-0.001	0.007	0.950
rs1373178	18	Intron:DCC	T	G	0.41	0.020	0.003	4.16E-15	0.41	-0.010	0.007	0.120
rs2359180	18	Intergenic	A	G	0.63	0.014	0.003	4.98E-08	0.73	0.002	0.010	0.840
rs34342129	18	Intergenic	T	C	0.49	0.014	0.003	2.13E-08	0.49	0.004	0.006	0.580
rs4476253	18	Intergenic	G	A	0.76	0.019	0.003	5.78E-10	0.76	0.012	0.008	0.110
rs62098013	18	Intron:DCC	A	G	0.37	0.018	0.003	2.24E-11	0.37	-0.001	0.007	0.900
rs67050670	18	Intergenic	A	G	0.77	0.020	0.003	2.34E-11	0.77	0.017	0.008	0.029
rs71367544	18	Intergenic	T	C	0.20	0.021	0.003	8.54E-11	0.20	0.020	0.008	0.012
rs72898831	18	Intergenic	A	G	0.85	0.024	0.004	4.14E-12	0.85	0.028	0.009	0.002
rs72938304	18	Intergenic	G	A	0.89	0.027	0.004	1.36E-11	0.88	-0.023	0.010	0.021
rs7505855	18	Intron:NOL4	C	T	0.41	0.017	0.003	5.31E-11	0.41	-0.010	0.007	0.130
rs8083764	18	Intron:DCC	G	T	0.69	0.016	0.003	7.97E-09	0.69	-0.014	0.007	0.044
rs8096225	18	Intron:MIR924HG	C	A	0.70	0.016	0.003	2.63E-08	0.70	0.012	0.007	0.100
rs10853981	19	Intergenic	A	G	0.33	0.015	0.003	4.88E-08	0.33	0.028	0.007	0.000
rs1126757	19	Synonymous:IL11	T	C	0.47	0.014	0.003	2.92E-08	0.48	-0.005	0.007	0.480
rs113230003	19	Intron:PGPEP1	G	A	0.75	0.019	0.003	1.05E-10	0.75	0.012	0.008	0.130
rs117734003	19	Intron:SYT3	C	G	0.07	0.030	0.005	2.57E-09	0.07	0.030	0.013	0.021
rs76608582	19	Intron:HDGFL2	C	A	0.95	0.035	0.006	4.88E-09	0.95	0.013	0.016	0.440
rs8103660	19	Intron:ELL	C	T	0.35	0.016	0.003	3.03E-09	0.36	-0.004	0.007	0.590
rs1555445	20	Upstream:NOL4L-DT	T	A	0.32	0.019	0.003	7.75E-12	0.32	0.005	0.007	0.450
rs3810496	20	Intron:ZBTB46	C	T	0.62	0.016	0.003	1.54E-09	0.62	0.019	0.007	0.004
rs6011779	20	Intron:CHRNA4	C	T	0.19	0.019	0.003	2.83E-09	0.19	0.013	0.009	0.120
rs6050446	20	Nonsynonymous:ENTPD6	G	A	0.97	0.054	0.008	8.80E-13	0.97	0.043	0.019	0.022
rs6058782	20	Intergenic	T	C	0.91	0.030	0.004	1.78E-11	0.91	0.011	0.011	0.340

rs6073075	20	Intergenic	T	A	0.18	0.019	0.003	2.44E-08	0.17	0.008	0.009	0.350
rs910912	20	Intergenic	T	C	0.26	0.017	0.003	7.82E-09	0.26	-0.004	0.007	0.590
rs4818005	21	Intron:BRWD1	G	A	0.42	0.020	0.003	1.09E-14	0.42	-0.017	0.006	0.010
rs139896	22	Intron:POLR2F	C	T	0.65	0.015	0.003	7.14E-09	0.65	0.017	0.007	0.012
rs4822102	22	Intergenic	C	T	0.38	0.017	0.003	2.78E-10	0.39	-0.006	0.007	0.360
rs9627272	22	Intergenic	G	C	0.59	0.0155	0.0026	2.42E-09	0.60	-0.007	0.007	0.310

Chr indicates chromosome; EA, effect allele; EAF, effect allele frequency; OA, other allele; SE, standard error; SNP, single-nucleotide polymorphism.

Supplementary Table 3. Association of smoking initiation with type 2 diabetes using dataset with body mass index adjustment

Method	OR (95% CI)	P
IVW-random effects	1.13 (1.06, 1.21)	1.18E-04
Weighted median	1.17 (1.08, 1.25)	3.57E-05
MR-Egger	1.05 (0.81, 1.37)	0.697
MR-PRESSO (5 outliers corrected)	1.13 (1.07, 1.20)	4.52E-05
I^2 (%)	50 (44, 56)	<0.001

CI indicates confidence interval; OR, odds ratio.

Supplementary Figure 1. Scatter plot for the association between smoking initiation and type 2 diabetes

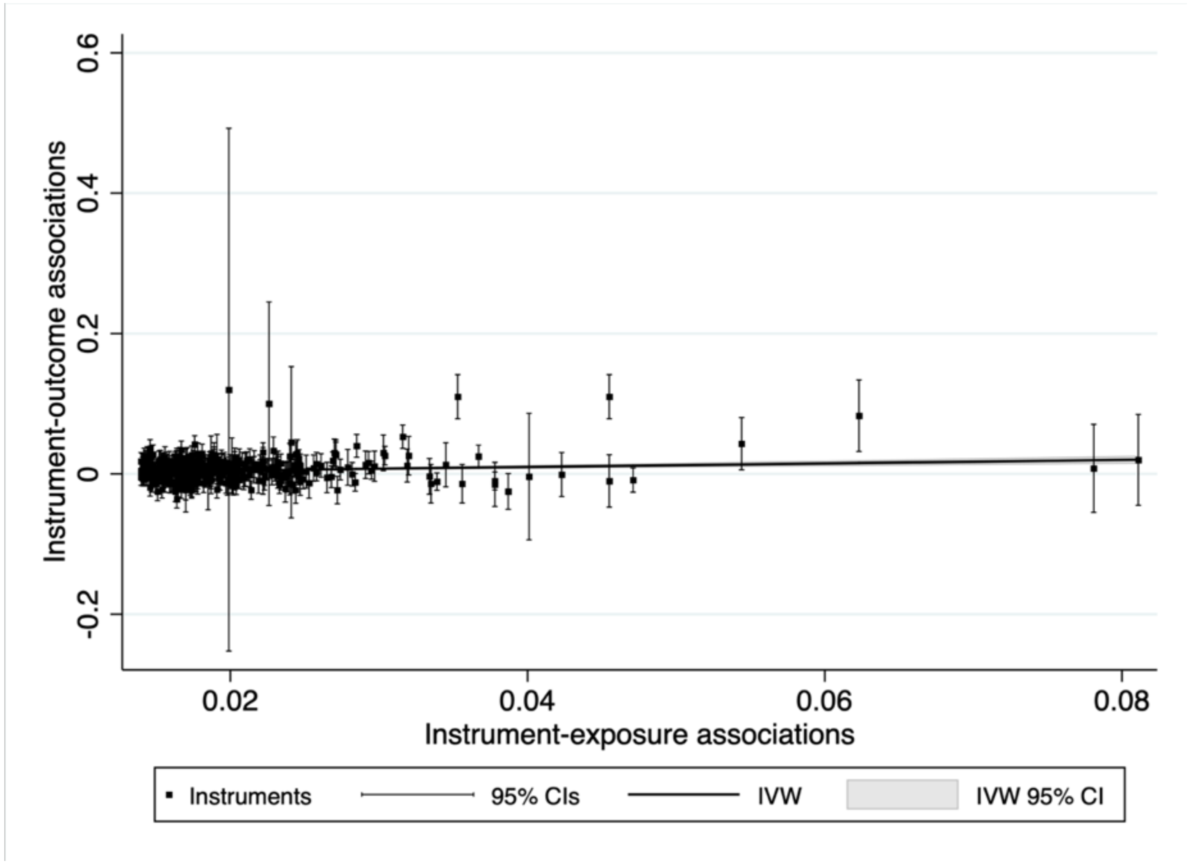

The grey bars indicate 95% confidence intervals. The solid line represents the inverse variance weighted estimate.
